# Human Adipose-Derived Stem Cell Secreted Extracellular Matrix Incorporated into Electrospun Poly(Lactic-*co*-Glycolic Acid) Nanofibrous Dressing for Enhancing Wound Healing

**DOI:** 10.3390/polym11101609

**Published:** 2019-10-03

**Authors:** Kao-Chun Tang, Kai-Chiang Yang, Che-Wei Lin, Yi-Kai Chen, Ting-Yu Lu, Hsien-Yeh Chen, Nai-Chen Cheng, Jiashing Yu

**Affiliations:** 1 Department of Chemical Engineering, College of Engineering, National Taiwan University, Taipei 106, Taiwan; b00701230@gmail.com (K.-C.T.); willy0511@hotmail.com (C.-W.L.); sleepykai72@gmail.com (Y.-K.C.); bbb32037@gmail.com (T.-Y.L.); hsychen@ntu.edu.tw (H.-Y.C.); 2 School of Dental Technology, Taipei Medical University, Taipei 106, Taiwan; pumpkin@tmu.edu.tw; 3 Department of Surgery, National Taiwan University Hospital, Taipei 100, Taiwan; naichenc@gmail.com

**Keywords:** wound healing, electrospinning, extracellular matrix, human adipose-derived stem cells

## Abstract

Wound dressing, which prevents dehydration and provides a physical barrier against infection to wound beds, can improve wound healing. The interactions between extracellular matrix (ECM) and growth factors is critical to the healing process. Electrospun nanofibers are promising templates for wound dressings due to the structure similarity to ECM of skin. Otherwise, the ECM secreted by human adipose-derived stem cells (hASCs) is rich in growth factors known to enhance wound healing. Accordingly, we propose that the PLGA nanofibrous template incorporated with hASCs-secreted ECM may enhance wound healing. In this study, PLGA nanofibrous matrixes with an aligned or a random structure were prepared by electrospinning. Human ASCs cultured on the aligned matrix had a better viability and produced a larger amount of ECM relative to that of random one. After 7 days’ cultivation, the hASCs on aligned PLGA substrates underwent decellularization to fabricate cECM/PLGA dressings. By using immunohistochemical staining against F-actin and cell nucleus, the removal of cellular components was verified. However, the type I collagen and laminin were well preserved on the cECM/PLGA nanofibrous matrixes. In addition, this substrate was hydrophilic, with appropriate mechanical strength to act as a wound dressing. The L929 fibroblasts had good activity, survival and proliferation on the cECM/PLGA meshes. In addition, the cECM/PLGA nanofibrous dressings improved the wound healing of surgically created full-thickness skin excision in a mouse model. This hASCs-secreted ECM incorporated into electrospun PLGA nanofibrous could be a promising dressing for enhancing wound healing.

## 1. Introduction

Wound healing is a complex process that includes the hemostasis, inflammation, proliferation, and remodeling stages. Despite skin having a self-regenerative capacity, wound dressings can promote absorption of exudates, prevent microbial infection, and improve tissue repair [1]. The wound dressings, which can be classified into traditional dressings, biomaterial-based dressings and biologic dressings, have been widely used in clinic [2]. By providing native skin structure and recruiting precursor cells, a biomimic and bioactive wound dressing can even enhance the healing process for large lesions and chronic wounds [3].

Electrospun nanofibers are promising wound dressings due to their structural similarity to the extracellular matrix (ECM) of skin [4]. The nanofibrous membranes, which have the characteristics of a high surface-to-volume ratio and high porosity, can promote cell adhesion and migration to improve tissue regeneration [5]. In addition to their superior mechanical properties [6], electrospun nanofibers provide good oxygen permeability, prevent dehydration of wound beds [7], and establish an effective barrier against microorganisms. Otherwise, nanofibers may also serve as drug carriers for antibiotics and growth factors [8]. Natural biomaterials such as collagen [9] and chitosan [10], and synthetic biomaterials such as polyvinyl alcohol (PVA) [11] and polylactic acid (PLA) [12] have been used to successfully fabricate nanofibers. Other research groups have further functionalized biomolecule-grafted nanofibers for specific applications such as osteogenesis and cardiac tissue engineering [13,14], revealing that a physiologically relevant biointerface could facilitate tissue regeneration.

Decellularized ECM, whether fabricated from tissues harvested in vivo or cell cultivation in vitro, is an appropriate ECM model because of the native structure and composition [15]. Decellularized ECMs have been used to facilitate tissue remodeling and reconstruction in a variety of clinical applications [16]. Cell-secreted ECM (cECM) can be derived directly from the cultivation of cells in vitro [17], and the decellularization can reduce immunogenicity [18,19]. Different types and abundant amounts of cECM can be produced for the fabrications of 2D [20,21] substrates or 3D scaffolds [22]. Furthermore, cell culture-secreted cECM provides an ideal ECM substitute, which can eliminate the difficulty of tissues shortage and harvesting.

Human adipose-derived stem cells (hASCs) are used in numerous regenerative applications due to the easy accessibility and a well-established isolation procedure [23,24]. The hASCs-secreted ECM is rich in growth factors including basic fibroblast growth factor (bFGF), transforming growth factor-beta (TGF-β), and vascular endothelial growth factor (VEGF) [25,26]. The interactions between ECM and growth factors is critical to wound healing [27]. Other studies have reported that these growth factors improve cell proliferation and migration to fibroblasts during the proliferation phase of wound healing [28,29]. Poly(lactide-co-glycolide) (PLGA), an FDA-approved biocompatible and biodegradable material, has been fabricated as nanofibers and applied in bone regeneration [30], cartilage reconstruction [31], nerve injury repairing [32], and wound healing [33,34]. The lactide:glycolide ratios affect both the in vitro and in vivo degradation rate of the PLGA scaffold materials due to the autocatalytic effect of the accumulated acidic degradation products surrounding the implants. Both the compositions of the PLGA polymers and the environmental conditions influence the degradation and tissue repair [35,36,37].

In this study, we proposed that the PLGA nanofiber incorporated with hASCs-secreted ECM may enhance wound healing. A mouse fibroblast cell line was used to assess the biocompatibility of hASCs-derived ECM/electrospun PLGA nanofibrous mesh. Finally, a mouse full-thickness wound model was used to verify effects of this bioactive dressing on wound healing.

## 2. Materials and Methods

### 2.1. Electrospinning of PLGA Nanofibrous Template

**Figure 1:**
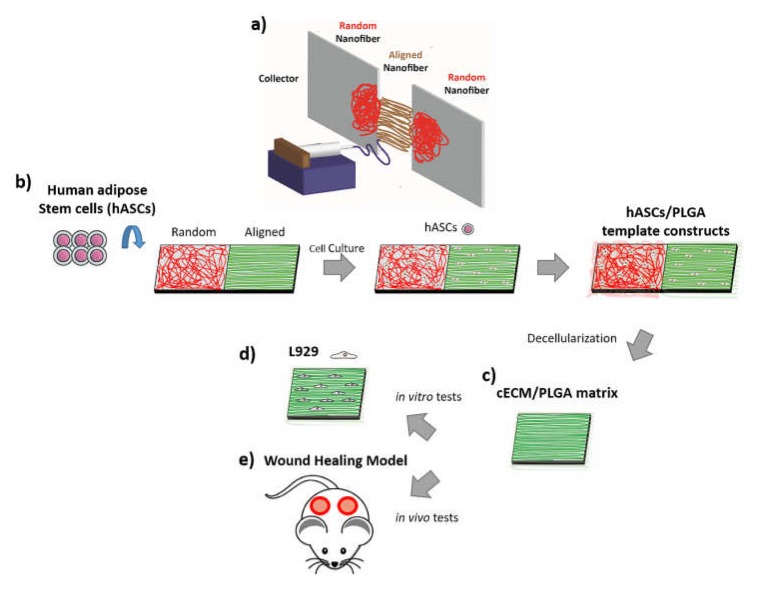
(**a**) Illustration of electrospinning system for PLGA nanofibrous tempate. (**b**) Human ASCs were seeded on the PLGA nanofibrous matrixes with either an aligned or a random structure. (**c**) The hASCs/PLGA nanofibrous meshes were decellularized to prepare cECM/PLGA matrixes. (**d**) Mouse L929 fibroblasts were seeded on the eECM/PLGA matrixes for in vitro study. (**e**) A surgical created full-thickness skin excision mouse was used as an in vivo model.

The electrospinning system comprised a high-voltage supply, an injection applause, and a grounded dual-plate collector (Figure 1a). The PLGA granules (85:15 poly(dl-lactide-co-glycolide, P85DG140, Green Square Materails Inc., Taiwan) were first dissolved in 1,1,1,3,3,3-hexafluoro-2-propanol (HFP, 105228, Sigma-Aldrich, St. Louis, MI, USA) solvent at 8% *w/v* concentration, which was then loaded into a syringe and applied to the syringe pump (Model 100, KD Scientific, Holliston, MA, USA) of the injection applause [21]. A 25-gauge metal needle with a 0.514 mm inner diameter was connected to the syringe for jet initiation. The flow rate of the PLGA solution was set at 4 mL/h with a 16,000 V supply (HVPS, YOU-SHANG, Taoyuan, Taiwan), and the distance between the tip of the needle and the collector was 10 cm. The collection time was 15 min, in which random nanofibers were collected on the surface of two plates or aligned nanofibers were produced within the gap between the two plates. Finally, the nanofibrous matrices were removed from the collector surface and placed in a 30 °C oven for 24 h to remove the residual HFP solvent. 

### 2.2. Characterizations of Electrospun PLGA Nanofibers

The microstructure of PLGA nanofibrous meshes with either a random or an aligned structure was observed using a scanning electron microscope (SEM, NovaTM NanoSEM 23, FEI, Milpitas, CA, USA). The samples were fixed with 2.5% glutaraldehyde solution (50-00-0, ACROS Organics, Belgians, WI, USA) at room temperature for 2 h and dehydrated in graded ethanol series. Finally, the samples were critical point dried, sputter-coated with gold ions for 60 s and investigated by the SEM. 

The degree of alignment of electrospun PLGA nanofiber was determined by using the software Image J (bundled with Java 1.8.0_112, Windows 10/XP) with the Fast Fourier Transform (FFT) function. For the analytical process, SEM images in 1500× magnification were selected and converted to an integer power of 2 pixel dimensions (512 × 512) and greyscale (8-bit) for 2D FFT analysis. When the Image J oval profile plugin was implemented, the pixel intensity along the radius at each angle was summed and the summed intensity was plotted against the corresponding angle to produce a 2D FFT alignment plot.

### 2.3. Cultivation of hASCs on PLGA Matrix

The isolation of hASCs was conducted as a previous study [38]. The hASCs were cultured in the DMEM:Ham’s F12 (DMEM/F12, SH30004.02, HyClone, St. Louis, MI, USA) supplemented with 1 ng/L human HGF, 10% (*v/v*) fetal bovine serum (FBS, 04-001-1A, Biological Industries, Cromwell, CT, USA), and 1% antibiotic (A5955, Sigma-Aldrich, St. Louis, MI, USA) in an incubator setting at 37 °C, 5% CO_2_ and saturated humidity. After reaching 70–80% confluence, the hASCs were detached and sub-cultured.

Electrospun PLGA nanofibrous meshes with either an aligned or a random structure were first fit into the 24-well tissue culture plates, and subsequently seeded with hASCs at a density of 2 × 10^5^ cells/well. The hASCs/PLGA nanofibrous meshes were also cultured in the DMEM/F12 medium, and the medium was changed every 2–3 days (Figure 1b).

### 2.4. AlamarBlue Assay for Cell Viability

After 1-, 4- and 7-day cultivations, the cell viability of hASCs on PLGA nanofibrous meshes was determined by using the alamarBlue assay (AlamarBlueTM Cell Viability Reagent, DAL1025, Thermo Fisher scientific, Waltham, MA, USA). The same number of hASCs were also seeded into the tissue culture plate as a comparison. At pre-determined intervals, the culture medium was removed and the cells on meshes were washed twice with phosphate buffer saline (PBS). The alamarBlue solution was added to each well and cultured for additional 4h in the incubator. Finally, 100 μL of the solution was transferred into a 96-well plates, and the result was measured at the wavelength of 570 nm with a reference at 630 nm by a microplate reader (ELx800, BioTek, Winooski, VT, USA).

### 2.5. Fabrication of cECM/PLGA Matrix and Protein Quantification

After 7-day cultivation, the hASCs/PLGA meshes were frozen in a −80 °C refrigerator and thawed to room temperature for six cycles for decellularization. The hASCs/PLGA mesh constructs were washed twice with deionized water between every cycle. Finally, the samples were treated with 25 mM NH4OH (SO0335, Scharlau, Sentmenat, Barcelona) for 20 min to prepare cECM/PLGA nanofibrous matrixes (Figure 1c). 

The protein content of the cECM/PLGA nanofibrous matrixes was quantified by the bicinchoninic acid (BCA). The samples were treated with Triton X-100 (X198-07, J.T. Baker, Radnor, PA, USA) for 15 min before reacted with the BCA regent at 60 °C for 1 h. A standard curve of protein concentration was established using a sequential dilution of the bovine serum albumin (BSA) powders. The results of BCA assay was measured at the wavelength of 570 nm by a microplate reader.

### 2.6. Characterizations of cECM/PLGA Nanofibrous Matrix

For the fabrication of cECM/PLGA nanofibrous meshes for further study, hASCs were seeded on the aligned nanofibrous meshes with a cell density of 2 × 10^5^, 3 × 10^5^, or 4 × 10^5^ cells/mesh. After 7-day cultivation, hASCs-secreted ECM/PLGA PLGA nanofibrous matrixes were decellularized and prepared for SEM inspections as previous section. 

The cECM/PLGA nanofibrous mesh samples were also fixated in 3.7% formaldehyde solution for 15 min, permeated with 0.2% Triton X-100 solution for 10 min for permeabilization, and immersed in 5% BSA solution for 1 h to block non-specific binding. Finally, the samples were incubated with phalloidin staining solution for F-actin (500 nM, P1951, Phalloidin-Tetramethylrhodamine B isothiocyanate, Sigma-Aldrich, St. Louis, MI, USA) for 40 min, and the cell nuclei were stained with Hoechst 33342 solution (H21492, Thermo Fisher scientific, Waltham, MA, USA) for 1 min.

Other cECM/PLGA samples were underwent immunofluorescence (IFC) staining against primary anti-collagen type I (ab90395, abcam, Cambridge, MA, USA) or anti-laminin (L9393, Sigma-Aldrich, St. Louis, MI, USA) antibodies at 4 °C overnight. Secondary antibodies with fluorescent probes (Alexa Fluor^®^ 488 for green fluorescence, A11008, molecular probes, Eugene, OR, USA; Alexa Fluor^®^ 594 for red fluorescence, A11005, molecular probes, Eugene, OR, USA) were then used to conjugate with primary antibodies at room temperature for 1 h. Finally, the nuclei were stained with Hoechst 33342 for 1 min.

### 2.7. Characterization of the cECM/PLGA Nanofibrous Mesh

Attenuated total reflection Fourier transform infrared spectroscopy (ATR-FTIR, Spectrum 100, Perkin Elmer, Waltham, MA, USA) was used to analyze the cECM/PLGA samples. After the mesh was freeze-dried, the spectra of the samples were taken between 4000 cm^−1^ and 650 cm^−1^.

For the measurement of water contact angle, the PLGA or cECM/PLGA nanofibrous mesh samples were placed on a glass slide on the stage. A drop of deionized water was added to the sample with a microsyringe. The images were captured for the analysis of contact angle (Contact angle system, FTA125, First Ten Angstroms, Cambridge, UK).

The tensile stress of cECM/PLGA nanofibrous samples was measured with an Elastic Modulus Load Cells (LTS-200GA, Kyowa, Hokkaido, Japan) and a Stepping Motor Driven Stages (SGSP(MS)20-85, Sigma Koki, Tokyo, Japan). The mesh was freeze-dried before tensile testing. The machine was set to stretch at a stretching velocity of 100 μm/s. The stress-displacement curve was then determined and analyzed by continuous stress recording.

### 2.8. Cultivation of Mouse Fibroblast L929 and Seeding onto cECM/PLGA Meshes

The mouse fibroblast cell line L929 cells (NCTC clone 929 of strain L) were cultured in DMEM/F12 medium supplemented with 2mM L-glutamine, 10% (*v/v*) FBS, and 1% antibiotic. The fibroblasts were seeded onto the cECM/aligned PLGA nanofibrous meshes at a cell density of 1.5 × 10^4^ cells/mesh in the 24-well tissue culture plates (Figure 1d). The fibroblasts/cECM/PLGA mesh constructs were cultured in regular DMEM/F12 medium, and the medium was changed every 2–3 days.

### 2.9. Evaluations of L929 Fibroblasts on cECM/PLGA Nanofibrous Mesh

After culturing for 1, 4, and 7 days, the L929 fibroblasts on cECM/PLGA nanofibrous meshes were subjected to n IFC staining procedure for the F-actin (phalloidin) and cell nucleus (Hoechst 33342), as in the previous section. In addition, the cell survival of L929 fibroblasts on the cECM/PLGA meshes was analyzed. At predetermined time points, the culture medium was removed, and the cells were washed twice with PBS. The samples were subjected to staining (LIVE/DEADTM Viability/Cytotoxicity Kit, L3224, molecular probes, Eugene, OR, USA) in a solution containing 5 μL calcein AM and 20 μL Ethidium homodimer-1 (EthD-1) in 10 mL PBS for 40 min. Finally, the cell viability of L929 fibroblasts on cECM/PLGA nanofibrous meshes was also determined by using the alamarBlue assay.

### 2.10. Wound Healing Animal Model

The animal experiments were approved by the Institutional Animal Care and Use Committee of the National Taiwan University (code: 002). A total of thirty ICR (CD1) mice (male, weight 29–32 g, aged 8 weeks; BioLASCO, Taipei, Taiwan) were used. The establishment of wound healing animal model was revised from a previous study [39]. In brief, the surgical procedure was conducted under general anesthesia. After adequate skin preparation and sterilization, bilateral full-thickness excisions (2 cm^2^ in size) were made on the dorsal skin of mouse using a sterile punch (Figure 1e). After that, the cECM/PLGA or PLGA nanofibrous meshes were covered the wound bed without suture. Mice that received bilateral full-thickness skin excisions without further treatment were served as a control group. The surgical created wounds with/out treatments were observed and recorded with a dissecting microscope photography system at day 1, 4, 7, 10 and 14 post-operation. The size of wound bed was measured using the Image J software, and the percentage of wound closure was calculated by comparison to the wound area at day 0.

### 2.11. Histology and Immunofluorescence Staining

All mice were sacrificed at day 14, and the repaired skin tissues including surrounding wound margins were harvested. The samples were fixed in 10% formalin with a neutral buffer, dehydrated, and embedded in paraffin wax. The paraffin blocks were cut into 5-μm slides in consecutive sections. The sections were further deparaffinized and stained with hematoxylin and eosin (H&E, 30721, Honeywell, Charlotte, NC, USA).

### 2.12. Statistical Analysis

All results are expressed as mean ± standard deviation of the mean (SD). Comparisons between different groups were analyzed by Student’s *t*-test, and the difference was considered significant when the *p*-value was less than 0.05.

## 3. Results and Discussion

### 3.1. Evaluation of Nanofibers with Different Orientations for hASCs/PLGA Meshes

**Figure 2:**
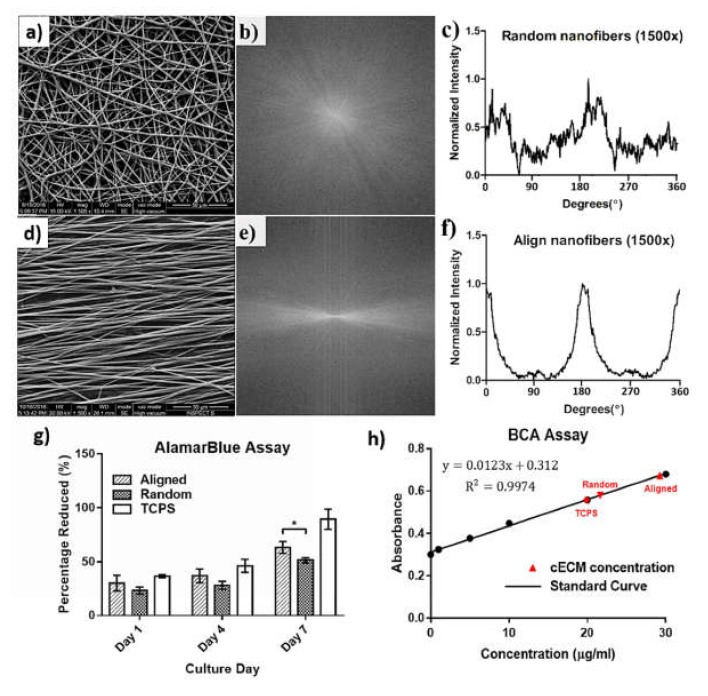
Characterizations of electrospun PLGA nanofiber. The PLGA nanofiber matrix with (**a**) a random or (**d**) an aligned structure was observed unser SEM. (**b**,**e**) SEM images of nanofibers were converted to an integer power of 2 pixel dimensions and greyscale for 2D FFT analysis. (**c**) No regularity of fiber alignment in random mesh samples. (**f**) Two peaks at 180° and 360° were found on the plot of the aligned nanofibers. The hASCs on the aligned PLGA nanofibrous meshes had (**g**) a significantly higher viability (*p* < 0.05) and (**h**) a higher amount of ECM content relative to that of the random ones at day 7.

Electrospun PLGA matrixes with random nanofibers (Figure 2a) or aligned structure (Figure 2d) were observed under SEM. The diameter of a single fiber ranged between 3 and 4 μm. The results of FFT analysis showed the orientations of fiber alignment (Figure 2b,e). There was no regularity of fiber alignment in random mesh samples (Figure 2c). On the contrary, two peaks at 180° and 360° were found on the plot of the aligned nanofibers (Figure 2f). 

For the alamarBlue assay, hASCs on the aligned PLGA nanofibrous meshes had a significantly higher viability relative to that of the random ones at day 7 (*p* < 0.05, Figure 2g). However, no significant differences were noticed in cell activity at day 1 and 4 between 2 groups. The protein quantification by BCA assay further showed that the ECM contents on aligned nanofibers (29.23 ± 0.95 μg/mL) was higher than that of the random ones (21.65 ± 0.76 μg/mL, Figure 2h). Therefore, aligned PLGA nanofibrous meshes were selected for the following study.

### 3.2. Staining of the cECM/PLGA Nanofibrous Mesh

**Figure 3:**
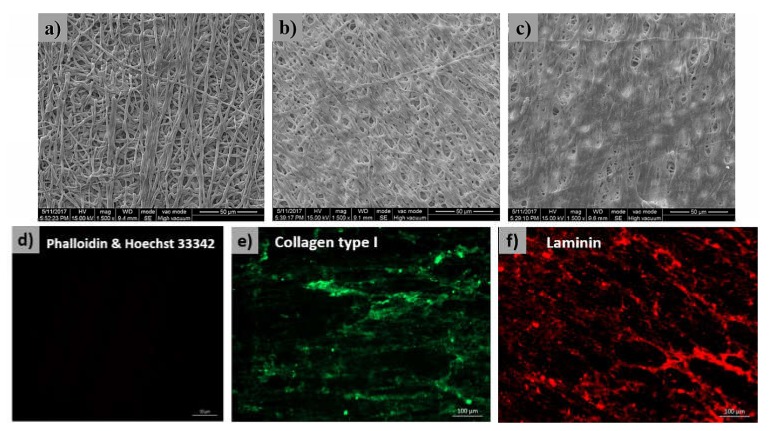
Surface morphology of cECM/PLGA templates showed that a thick ECM was found on the PLGA nanofibers. When the cell seeding density increased, a relative larger amount of ECM was also obtained (**a**) 2 × 10^5^, (**b**) 3 × 10^5^, and (**c**) 4 × 10^5^ cells/mesh. (**d**) The phalloidin staining against cytoskeleton F-actin and Hoechst 33342 staining against cell nucleus proved there was no remaining cytoskeleton or cell nucleus in the cECM/PLGA templates. IFC staining revealed that the ECM components (**e**) collagen type I and (**f**) laminin were well preserved.

The morphology of cECM/PLGA nanofibers was shown in Figure 3a–c. Relative to the blank nanofibrous mesh, a thick ECM was found on the cECM/PLGA nanofibers under SEM observation. When the cell seeding density increased, a relative larger amount of ECM was also obtained (Figure 3a for 2 × 10^5^, Figure 3b for 3 × 10^5^, and Figure 3c for 4 × 10^5^ cells/mesh). Therefore, the cell density 4 × 10^5^ cells/mesh was used in the following study.

To verify the decellularization procedure, phalloidin staining against cytoskeleton F-actin and Hoechst 33342 staining against cell nucleus were conducted. No cytoskeleton or cell nucleus was found (Figure 3d), which revealed that the decellularization was complete. On the other hand, IFC staining showed a large quantity of collagen type I (green fluorescence, Figure 3e) and laminin (red fluorescence, Figure 3f) was preserved in the cECM/PLGA nanofibers, which was consistent with the findings of the SEM inspection. 

### 3.3. Characterization of the cECM/PLGA Nanofibrous Mesh

**Figure 4:**
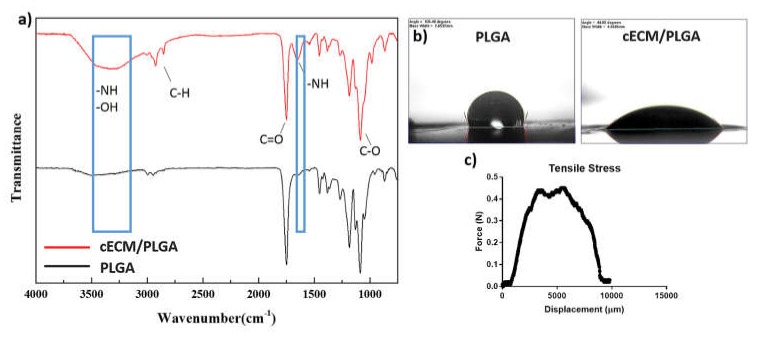
(**a**) FT-IR spectrum showed the presences of −NH and −OH groups (3300 cm^−1^), and another peat at 1600 cm^−1^ also represented –NH group. (**b**) The test of water contact angle showed a hydrophilic surface on the cECM/PLGA substrates. (**c**) The tensile stress test showed that the cECM/PLGA mesh had the highest stress tolerance around 0.5 N.

The FTIR spectrum of cECM/PLGA samples indicated the presences of −NH and −OH groups (3300 cm^−1^), and another peat at 1600 cm^−1^ also represented –NH group. However, no relevant peaks were found in the PLGA meshes (Figure 4a). Regarding the hydrophilicity, PLGA showed a hydrophobic property (Figure 4b). The water contact angle of cECM/PLGA mesh was 113.25° ± 1.03°, which represented a hydrophilic surface. The tensile stress test of cECM/PLGA sample showed the highest stress tolerance was about 0.5 N (Figure 4c), which may provide appreciate mechanical strength as a wound dressing.

### 3.4. Evaluations of Fibroblasts on cECM/PLGA Nanofibrous Mesh

**Figure 5:**
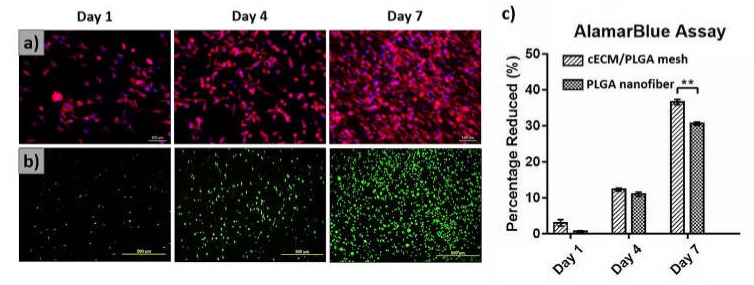
The L929 fibroblasts proliferated well on the cECM/PLGA matrixes with (**a**,**b**) good cell survival. (**c**) The cells on the cECM/PLGA meshes also had a relatively higher cell viability (*p* < 0.01) than those of one PLGA nanofibers at day 7.

The IFC staining against cytoskeleton F-actin (red fluorescence) and cell nucleus (blue fluorescence) revealed that the L929 fibroblast proliferated well on the cECM/PLGA nanofibrous mesh, and the number of cells increased with time (Figure 5a). Live/dead staining further revealed the cells had good survival (green fluorescence) through the experiential periods (Figure 5b). The results of alamarBlue assay further showed that L929 fibroblasts on the cECM/PLGA meshes had a relatively higher cell viability at day 7 (*p* < 0.01, Figure 5c).

### 3.5. Wound Healing Animal Study

**Figure 6:**
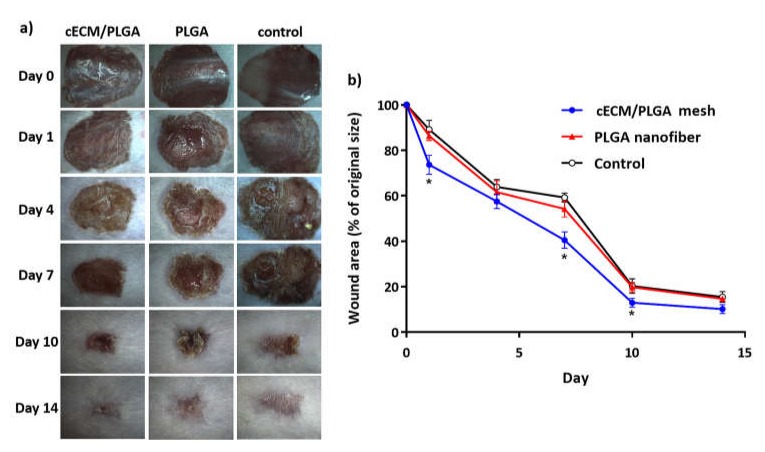
Wound healing model in mouse. (**a**) The gross observations revealed that all surgical created wound beds closed as time. However, the use of a PLGA nanofibrous mesh did not improve wound closure when compared to that of untreated wounds (control). On the contrary, the cECM/PLGA mesh enhanced wound healing, and the wound was almost closed at day 14. (**b**) The decrease in wound area indicated that mice received the cECM/PLGA meshes had a significant smaller wound size while there was no obvious difference between PLGA nanofiber and control group.

The gross observations of surgical created wound beds with/out treatments were shown in Figure 6a. All wound closed as time. However, the use of a PLGA nanofibrous mesh did not improve wound closure when compared to that of untreated wounds (control). The cECM/PLGA mesh enhanced wound healing, and the wound was almost closed at day 14. On the contrary, untreated mice and mice received the PLGA mesh showed a relatively slow wound healing process. The decrease in wound area indicated that mice received the cECM/PLGA meshes had a significant smaller wound size while there was no obvious difference between PLGA nanofiber and control group (Figure 6b).

## 4. Discussion

A wound dressing can prevent dehydration and provide a physical barrier against infection to wound beds. Biologic wound dressings, such as amniotic and placental membranes, are known to promote wound healing and used in clinical practices such as diabetic foot ulcers and burns [40]. Despite the efficacy and safety of these commercial biologic wound dressings being well accepted, the tissue source is still a limitation. Otherwise, several studies have studied the influence of cECM on wound healing [41,42]. Therefore, a cell culture-derived ECM product in combination with electrospun PLGA nanofibrous mesh is proposed as a novel wound dressing. 

In this study, electrospun PLGA nanofibers with either random structure (Figure 2a) or aligned geometry (Figure 2d) were fabricated. Despite Meng et al. finding that the orientations of PLGA nanofibrous scaffolds did not change the viability of mouse MC3T3 (an osteoblast precursor cell line) [43], another study reported that an aligned collagen nanofibers improved cell activity to neural progenitor cells [44]. Furthermore, Ma et al. noticed that the aligned electrospun PLA nanofibers improved activity to bone marrow stromal cells [45]. These studies revealed that the raw materials of nanofibers, as well as the cell types, all influence the viability of cells cultured on the electrospun matrixes. We also found the alignment degree of nanofibers (Figure 2c,f) influenced cell viability of hASCs (Figure 2g). In addition, hASCs also produced a relatively larger amount of ECM on the aligned PLGA matrixes (Figure 2h). Therefore, the electrospun PLGA nanofibrous matrix with an aligned geometry was used as a template for the manufacture of decellularized ECM.

Since the cellular components have residual antigenicity to cause potential immunogenicity, decellularization is the critical procedure to increasing the clinical implementation [46]. The SEM inspections showed that our decellularization approach preserved plenty of cECM on the PLGA nanofibers (Figure 3a–c). Furthermore, the Hoechst 33342, as well as phalloidin staining (Figure 3d), confirmed that the cellular components were removed while the major ECM components collagen type I (Figure 3e) and laminin (Figure 3f) were well preserved on the nanofibrous matrixes. Otherwise, the FTIR spectrum confirmed the presences of −NH and −OH groups (Figure 4a), which provided solid evidence for the ECM components on the PLGA templates [47]. Furthermore, the hydrophilicity (Figure 4b), which can improve cell adhesion, and mechanical strength (Figure 4c) of the cECM/PLGA samples also support the applicability of the electrospun nanofibrous mesh a suitable niche to wound healing. 

The cultivation of L929 fibroblasts on the nanofibrous meshes further showed that cECM/PLGA matrixes support cell proliferation (Figure 5a) and survival (Figure 5b). Relative to that of PLGA nanofiber, the cECM also improved the cell viability of fibroblasts (Figure 5c). For the in vivo study, we showed the cECM/PLGA meshes improved the wound closure to the full-thickness skin excision in mouse relative to the use of PLGA nanofibers (Figure 6a,b). Similarly, Navone et al. also showed decellularized adipose mesenchymal stromal cells/silk fibroin substrate improved wound healing in the diabetic mice [48]. Sadeghi et al. also demonstrated that the modification of PLGA fibers with collagen coating enhanced the human dermal fibroblasts (HDF) and keratinocytes cell line (HaCat) proliferation [49]. 

In accordance to our hypothesis and the previous studies shown, the cECM/PLGA nanofibrous dressing could benefit wound healing.

## 5. Conclusions

In this study, PLGA nanofibrous matrixes with an aligned or a random structure were prepared by electrospinning. Human ASCs showed a better cell activity and produced a larger amount of ECM on the aligned PLGA matrix relative to that of the random one. By using IFC staining against F-actin and cell nucleus, the efficiency of the decellularization was verified. However, the ECM components, including type I collagen and laminin, were preserved on the PLGA substrates. This finding was further confirmed with the FTIR spectrum in which the presences of −NH and −OH groups on the cECM/PLGA nanofibrous matrixes. In addition, this hydrophilic substrate had appropriate mechanical strength for use as a wound dressing. The L929 fibroblasts had good activity, survival and proliferation on the cECM/PLGA meshes. Finally, the cECM/PLGA nanofibrous meshes improved the wound healing of surgical created full-thickness skin excision in mice. This hASCs-secreted ECM incorporated into electrospun PLGA nanofibrous could be a promising dressing for enhancing wound healing.
